# Statistics of the Popularity of Chemical Compounds in Relation to the Non-Target Analysis

**DOI:** 10.3390/molecules26082394

**Published:** 2021-04-20

**Authors:** Boris L. Milman, Inna K. Zhurkovich

**Affiliations:** 1 Institute of Experimental Medicine, Ul. Akad. Pavlova 12, 197376 Saint Petersburg, Russia; 2 Institute of Toxicology, Ul. Bekhtereva 1, 192019 Saint Petersburg, Russia; zhurkovich.i.k@toxicology.ru

**Keywords:** non-target analysis, chemical databases, chemical compounds, popularity indicators

## Abstract

The idea of popularity/abundance of chemical compounds is widely used in non-target chemical analysis involving environmental studies. To have a clear quantitative basis for this idea, frequency distributions of chemical compounds over indicators of their popularity/abundance are obtained and discussed. Popularity indicators are the number of information sources, the number of chemical vendors, counts of data records, and other variables assessed from two large databases, namely ChemSpider and PubChem. Distributions are approximated by power functions, special cases of Zipf distributions, which are characteristic of the results of human/social activity. Relatively small group of the most popular compounds has been denoted, conventionally accounting for a few percent (several million) of compounds. These compounds are most often explored in scientific research and are practically used. Accordingly, popular compounds have been taken into account as first analyte candidates for identification in non-target analysis.

## 1. Introduction

The idea that chemical compounds can be popular/abundant/prevalent/widespread or rare is widely used in modern analytical chemistry, namely in the non-target chemical analysis involving environmental studies [1,2,3,4]. An analytical chemist, considering the results obtained and comparing them with reference data, may come to an ambiguous conclusion about the nature of the detected compound, allowing several identification hypotheses regarding known unknowns. To obtain a reliable true result, it is necessary to find analytical standards corresponding to the candidate compounds and compare their features with the obtained experimental data. To optimize this work, it is advisable to reduce the number of candidates for identification and establish a procedure for verifying the corresponding versions. At the same time, it is necessary to select the most suitable compounds, whose presence in the samples under analysis is most probable, and the verification should begin with these prospective candidate analytes.

Those have been commonly referred to as the chemical compounds most widespread in the physical universe (natural and artificial substances and materials). This abundance results in the popularity of compounds, measured by information indicators/rates such as “citations” of particular compounds in the scientific literature and hits of related database searches [1,2,3,4]. The hit rate is considered as a measure of popularity. These indicators are important not only quantitatively, but also heuristically. A significant presence in databases means not only the widespread abundance of the corresponding compound, but also a perceptible chance that meaningful data on the preparation, use, and properties of this compound could be searched to posteriori estimate the trueness of its detection and identification. Concerning the current experimental data, those popularity rates and meaningful information constitute the so-called metadata.

Distinguishing of popular compounds subset means, obviously that the rest covers rare compounds. In the absence of the first principles that would determine the assignment of substances to these two groups, the division into popular and rare compounds is conditional and depends on chemical data statistics. Its most important but hidden component is a distribution of chemical compounds over the corresponding indicators of popularity. Obtaining such distributions would make it possible (a) to outline, with varying degrees of conventionality, the boundary between popular and rare compounds, (b) to use this information meaningfully in non-target chemical analysis, and (c) to solve related problems, such as, for example, dereplication [5] and the formation of mass spectra libraries [6] and chemical databases.

The statistics of the popularity of chemical compounds and related facts are fragmentarily presented in several articles and electronic resources. Some of the most popular molecules occur in Wikipedia [7]; in October 2020, data on 17885 compounds were observed. Frequently occurred compounds, which are substrates and products of organic reactions, have been identified; their distributions by molecular weight [8] and abundance [8,9] were given. The distribution of ChemSpider database entries over molecular weight was also derived [2]. The statistics of the most common fragments of known chemical compounds have been published, including the distribution of compounds over the number of different fragments [10,11]. Some other statistical data on common compounds can be found in chemical databases (see reviews [12,13]).

As far as we know, little is known about the distributions of known substances over their popularity, relating to the chemical space as a whole. To fill this gap is the principal task of this work, which includes determining the parameters of such distributions and, on this basis, estimating both the boundaries that distinguish popular compounds from other ones and the sizes of their subset (subspace). In addition, we wanted to outline some areas of application of our findings. The biggest non-commercial chemical databases such as ChemSpider [14] and PubChem [15] (Table 1) were chosen as the data sets required for such work. The first of these databases is of prime importance for mass spectrometry since it allows one to establish the molecular formulas of particular compounds by the masses of their ions. The second database contains extensive records of the biochemical and biological properties of many chemical compounds.

Rigorous conclusions about the features of objects/compounds presented in these databases can be made by analyzing their complete coverage or by a representative (random) sample of them (see [16]). Full simultaneous searches of popularity rates for all compounds are impossible for common users of these information systems, which do not have programmatic access. Therefore, the study was carried out on random samples of compounds.

Preliminary results of this research were partially published [17]. This article significantly expands the obtained data and corresponding conclusions. The authors hope that, based on these results, researchers will better use chemical databases in order, first, to estimate the abundance and degree of likelihood of candidates for identification in non-target analysis and, secondly, to generate their own data resources for solving various problems in this field of chemical analytics.

## 2. Methods

A random sample of chemical compounds is formed by their unambiguous identifiers, specifically identification numbers (IDs), in these databases using a random number generator [18]. Random IDs as integer numbers were generated in the range between 1 and maximum value, which was determined by searches in the databases (Table 1). The number of random IDs was chosen based on the reasonable level of corresponding statistical errors and some other aspects (see below). Random IDs are placed in Supplementary Materials, Table S1.

ChemSpider: Previously, we studied the features of this database [19], and therefore here we limited ourselves to a relatively small sample of 1000 IDs, for which 885 chemical compounds were found. The rest of 115 IDs turned out to be “empty”: there were no current compounds with corresponding ID in the database; those were obviously had been removed from this dataset. The most universal indicator characterizing the popularity/abundance of compounds is the number of data sources [4,19], i.e., the number of other databases from which information was extracted. A special case of this indicator is the number of vendors of these compounds; the information has been extracted from the relevant databases as electronic catalogs. First of all, these two indicators are determined.

An estimate of the number of scientific articles in which the corresponding compounds are cited would be important, but this is difficult to do since direct references to scientific articles and literary references available in the Google Scholar system were found only for a small fraction of the compounds (see below). Even less patent information was found. To some extent, the ChemSpider indicator of the total number of references (# of References) is important [2], which is visible, for example, when searching for compounds by a particular molecular formula or molecular weight. This indicator was estimated by the staff of the information system several years ago as a result of an external information search. The latter is not described with necessary details, and therefore its results were not explored here.

PubChem: Earlier [19] this database was studied by us in less detail. Therefore, a relatively big sample was formed, consisting of 50,000 IDs. In this subset, 35,069 (70%) chemical compounds were found; the rest of IDs was “empty”. The same indicators were obtained as in the case of the first database: the number of information sources and the number of chemical vendors. In addition, the number of scientific articles and the number of patents were determined. The indicators characterizing this particular database were also evaluated. They are the number of sections in the record for given compounds (according to the Contents section) and, most importantly, the conventional amount of information corresponding to the database record under consideration (Annotation Record Count). The last two indicators correlate well with each other.

Data sampling results in an appearance of a statistical error in the number and proportion of compounds with certain features (which are values of indicators). The error was estimated using the calculator on the website [20]. Specific error values are given in Table 2. Those reflect accuracy with which compound proportion in the sample reproduces the corresponding proportion in the original databases. For example, the observed proportions of compounds with the number of data sources equal to 1, 2, 3, 4, 5, and 6 in the ChemSpider sample were 58%, 19%, 5%, 3%, 3%, and 2%, respectively. In relation to the original ChemSpider database, these percentages were transformed into values of (58 ± 3)%, (19 ± 2)%, (5 ± 1)%, (3 ± 1)%, (3 ± 1)%, and (2 ± 1)%, respectively. With these accuracies/errors, there were significant differences in the first three rates and there were no such differences in the last three values. This is enough for correctness of the conclusion of our study, and the better population rates discrimination would be achieved by increasing the data sample, as in the case of PubChem (see Table 2).

## 3. Results and Discussion

**Figure 1:**
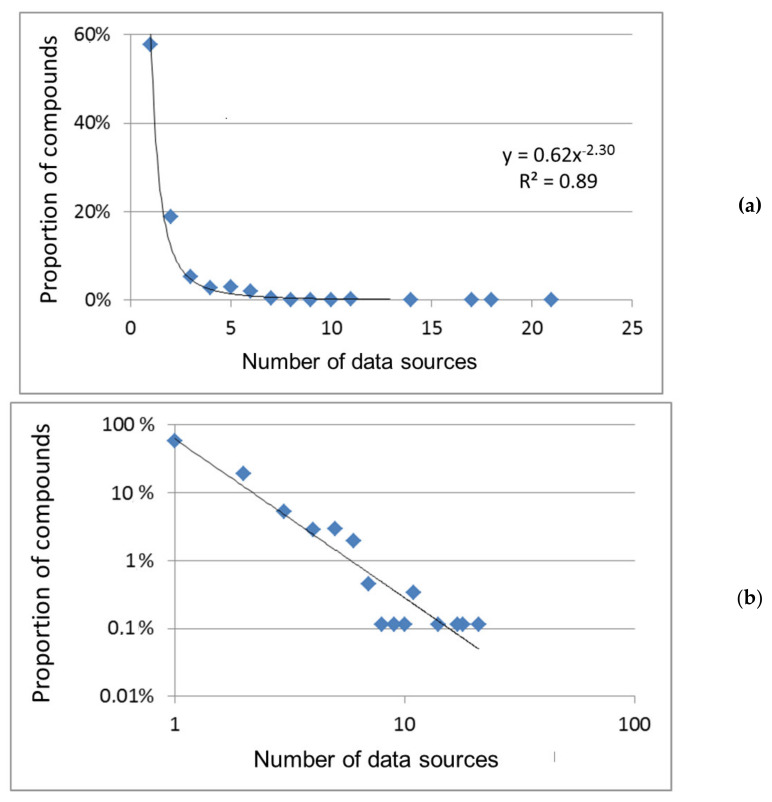

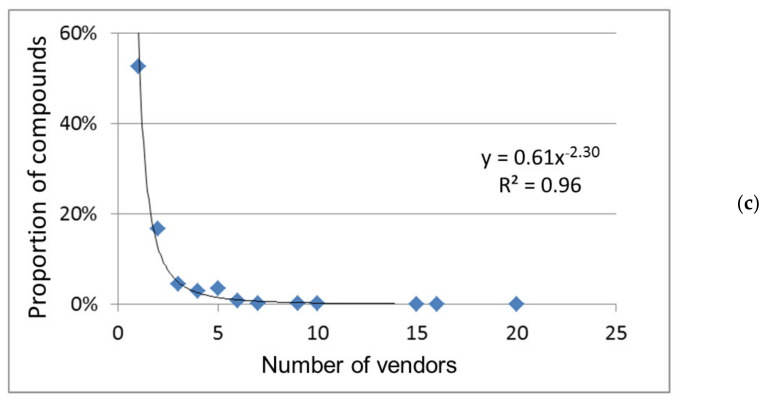
Distribution of compounds over different indicators in the ChemSpider database. (**a**) The number of sources. Indicators are absent (0) for (9 ± 2)% of compounds. (**b**) The same, logarithmic coordinates. (**c**) The number of vendors. Indicators are absent (0) for (17 ± 2)% of compounds.

**Figure 2:**
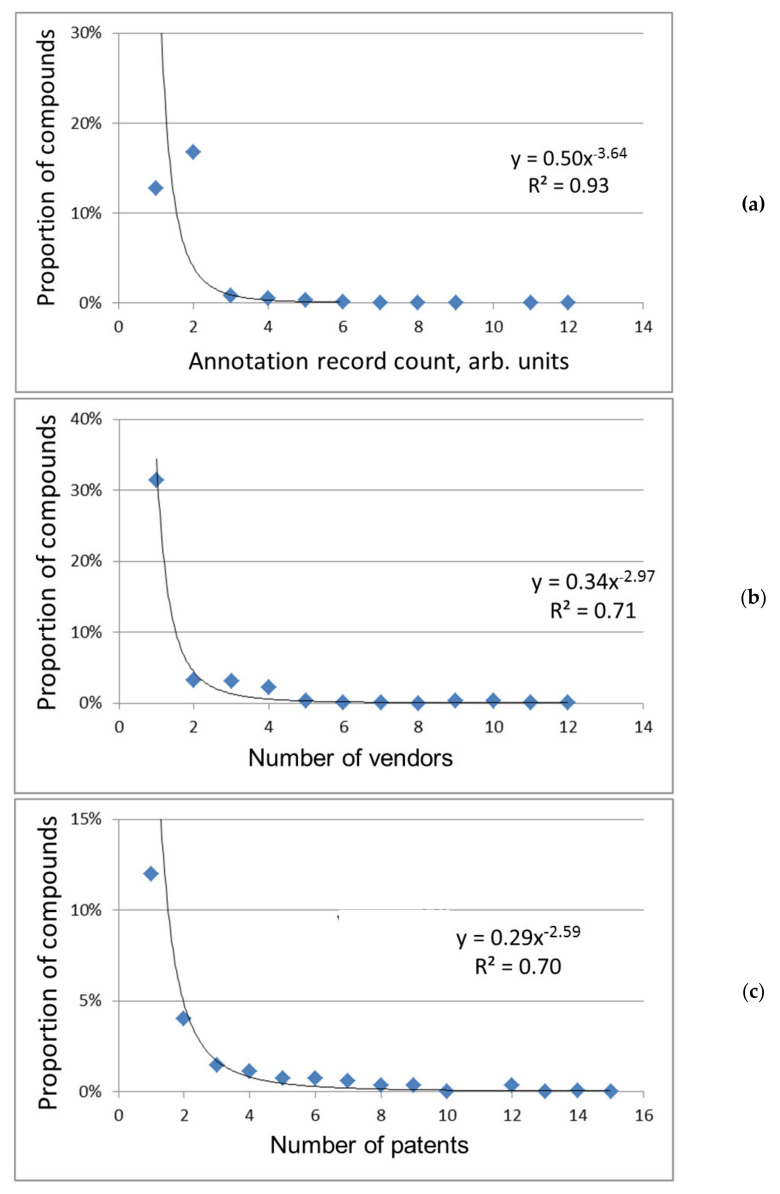

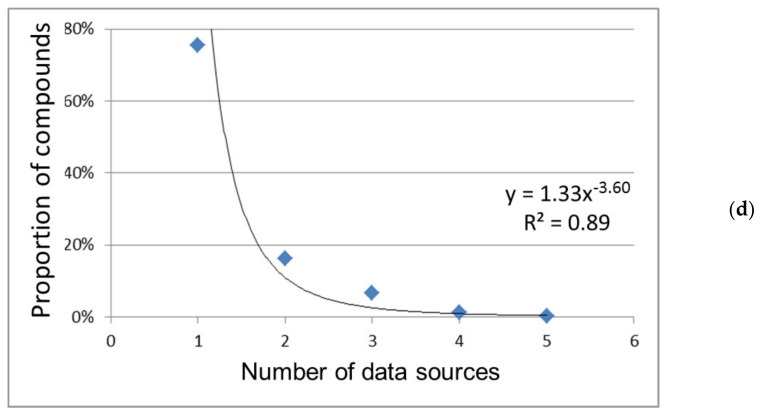
Distribution of compounds over different indicators in the PubChem database. (**a**) The size of records. Indicators are minimal (0) for (68.7 ± 0.4)% of compounds. (**b**) The number of vendors. Indicators are absent (0) for (59.1% ± 0.4%) of compounds. Very rare data for the most abundant compounds (from 14 to 75 vendors, total 0.1%) are not shown. (**c**) The number of patents. Indicators are absent (0) for (78.0% ± 0.4%) of compounds. Very rare data for the most abundant compounds (from 17 to 23036 patents, total 0.3%) are not shown. (**d**) The number of sources. Indicators are minimal (1, the database itself) for (75.4 ± 0.4)% of compounds. Very rare data for most abundant compounds (from 6 to 20 sources, total 0.3%) are not shown.

General regularities: The frequency distribution of chemical compounds over various indicators of their popularity is shown in Figure 1 and Figure 2. With an increase in indicator values, i.e., as the popularity of compounds grows, their proportion, in general, decreases. The dependences shown in the figures are well or adequately approximated by power functions with a negative exponent. Power dependences are a special case of Zipf distribution (non-Gaussian distribution) [21,22], common for social phenomena, various human activity, for example, for a system of science citation that is close in meaning to the citation of chemical substances under consideration. So, the popularity of chemical compounds is subject to the same general regularities as the citation of scientific publications i.e., is determined by the importance of compounds for a professional person in his/her research or practical activity.

The power functions in logarithmic coordinates are transformed into linear graphs (the example in Figure 1b). These graphs are common for compounds distribution over the frequency of their participation in organic synthesis [8,9] and for the types of molecular fragments distribution over the frequency of their presence in known compounds [10,11]. There are general regularities in such “chemical” frequency distributions, conditionally dividing chemical space into subspaces of more and less popular compounds. As for organic synthesis, the “addiction” of chemists to the same/widespread compounds and their corresponding conservatism were noted in the literature [9]. This is another manifestation of the Matthew effect [23].

In our work, the approximating power functions of different indicators of popularity differ from each other (Figure 1 and Figure 2), which may be a consequence of the discrepancy between these indicators in meaning and their different coverage of various groups of compounds. Thus, the number of patents reflects the current or prospective practical use of chemical compounds; new compounds not described in the scientific literature are often found in patent applications [24]. The number of references (see below) indicates the degree of involvement of compounds in scientific research. When it comes to supplying chemicals, they are purchased for different purposes: synthesis and manufacture, as analytical standards and basic or auxiliary substances in scientific experiments, etc. Therefore, the number of vendors in both databases is a fairly universal indicator of the abundance of chemical compounds, as well as the total count of information about them in the PubChem database and the number of sources of such information (both databases; ChemSpider is preferred, see below).

Attention can be drawn to the fact that most of the compounds in PubChem are not characterized by three indicators (i.e., their values equal to 0, Figure 2a–c). The indicator value of the number of sources equal to one can also be considered zero since the database here cites only itself (Figure 2d). Herewith, there is no or minimal information about the preparation, properties, and use of compounds (the count of such a record is conventionally equal to zero, Figure 2a). So, when comparing databases, the difference in the coverage and the number of relevant data sources used, the degree of data extraction from them, etc., are likely observed.

Common and rare compounds: Our preliminary research found that the most popular compounds are pharmaceuticals, amino acids, nucleobases, metabolites, and other scientifically and practically valuable bio compounds [17]. The newest version of the list of the most popular compounds confirms that conclusion (see Supplementary Materials, Table S2). However, most of the compounds of bio- and different origin are rare, with low popularity indicators (see Figure 1 and Figure 2). A rare compound set covers less important substances and new compounds that did not have time to score large popularity rates. It is important that the new compounds can be identified by their large IDs.

However, the classification of compounds as abundant or rare is conditional and depends on the choice of the boundary values of the indicators. In statistics, used, for example, in analytical chemistry, when evaluating the ranges of values of certain quantities, the probability values 0.90, 0.95, and 0.99 are commonly chosen [25]. With these probabilities, the main values (99%, 95%, and 90% of the sum, correspondingly) of quantities are located within the interval containing the maximum and outlined by the corresponding boundary values. These probabilities characterize Gaussian/normal distributions inherent in many physical and physicochemical properties of objects in the physical world. For non-Gaussian distributions, we will allow the same probabilities (0.90, 0.95, and 0.99 for rare compounds and, respectively, 0.10, 0.05, and 0.01 for popular compounds). These probabilities correspond to the proportion of rare compounds (99%, 95%, and 90%) with popularity rates in the range from minimal to the boundary and the proportion of abundant compounds (1%, 5%, and 10%) in the range from boundary to maximum.

The boundary values of the considered indicators for compounds of both groups are given in Table 3. The upper limit of indicators for the group of rare compounds is a small number of units (sources of information, vendors, patents, and counts of records), depending on the probability. This upper limit is the lower one of a relatively small group of popular compounds, indicators of which vary up to the specified maximum values.

In the proposed approach for estimating popularity, the choice of probability is rather arbitrary. Several possibilities for establishing a more definite boundary between popular and rare compounds can also be suggested.

1. Analytical chemists most often use a confidence probability of 0.95. With such a probability, 95% of the entire chemical space belongs to rare compounds, and 5% to popular ones, i.e., taking into account the coverage of these databases, several million compounds.
2. To estimate the number of rare and popular compounds, mismatched boundaries can be used: 90% for rare compounds, 1% (up to approximately one million) for popular ones. The intermediate interval of 90–99% (1–10%) may include representatives of both groups.

To identify a particular group of compounds, one can use other indicators that are not noted in Table 3. First of all, it is the number of scientific articles. Here we did not consider it as the main indicator. The reason is that the literature citing chemical compounds is available for only 1.7% (ChemSpider) and 2.5% (PubChem) compounds (according to our estimates). Such a small proportion of substances are due to the special aspects of the formation of these databases. It can be assumed that the search for compounds to be included in the database was carried out, first of all, by their text identifiers (InChI, SMILES), which are absent in common scientific articles (and in their electronic versions). In any case, with a nonzero number of literature sources available in these databases, a chemical compound can be considered popular/abundant. To best estimate these indicator values of the popularity of chemical compounds, it would make sense to use the Chemical Abstracts Service [26], which indexes all the chemical publications. In any case, the citation of chemical compounds in the scientific literature, calculated by the Chemical Abstracts reference journal, was the first indicator of the popularity of substances estimated for non-target analysis. For example, in 2003 the number of popular compounds (at least 10 citations in various articles) was relatively small and their proportion amounted to 1.3% [1].

Summing up the estimates of various indicators of popularity, it can be argued that the proportion of popular compounds is a few percent, and their number is no more than a few million.

Aspects of application: We will note again that recognition of a particular chemical compound as a popular/common one may be needed in various problems of non-target chemical analysis including identification of emerging substances of environmental concern. In general, several suitable popular compounds may be considered as candidates for identification with subsequent elimination of false identification hypotheses. What is important here is the relatively high popularity of a particular compound among a group of related compounds (with identical or similar molecular formulas/weights). The validation of the identification trueness using analytical standards (reference materials) can be started from this particular compound. In this case, the relative rather than absolute values of the popularity indicators may be more important.

Constantly or from time to time, the problem arises of database replenishment with some or other compounds. If the number of compounds covered by such a database is comparable to the total number of popular compounds, we can consider this database as complete enough and not requiring immediate upgrade. Note that it is also appropriate to update the information for established popular compounds, as demonstrated by the example of the reduced subset generated from PubChem [27].

A chemist knows that most chemical compounds are hazardous and that big environmental chemical databases should contain most of the popular compounds. So, for example, EPA CompTox Chemicals [28] covering 882 thousand compounds can be called a fairly complete database in the field of environmental research. In this context, we feel the incompleteness of modern mass spectral libraries for nonvolatile compounds (tandem mass spectra [6]), although recent progress in this field is observed. The today achievement probably belongs to the METLIN database covering more than 500 thousand compounds [29].

Another example of the use of ideas about the popularity of chemical objects is the search for new advanced natural compounds, for example, medicinal substances. Typically, mixtures of plant origin, potentially containing new beneficial compounds, are dominated by well-known molecules, discovered and characterized long ago as chemistry developed. The task of a modern researcher is dereplication that is the rapid identification of known compounds and their exclusion from consideration (including removal from mixtures) [5]. Big databases of popular chemicals should help to meet this challenge.

It is possible to imagine an analytical problem in which the prioritization strategy [30] would be the determination of all abundant chemical compounds. In this case, it is appropriate to generate a complete database of popular compounds with the size of subset derived here; the prototype of such work is available [27].

## 4. Conclusions

The article presents the frequency distributions of chemical compounds over the indicators of their popularity/abundance. The latter include the number of information sources, the number of chemical vendors and other variables, the values of which are taken from the big ChemSpider and PubChem databases. Distributions are well approximated by power functions belonging to the family of non-Gaussian/Zipf distributions, which describe the results of human activity including one of scientists, engineers, and technologists. Such distributions reveal a relatively small group of the most popular compounds, consisting of several million compounds. They are the most important for practical application and scientific research and therefore are characterized in the most detail; their popularity indicators are noticeably higher than corresponding average values. Information on popularity is primarily used in non-targeted chemical analysis when searching candidates for identification in a group of related compounds. It is also useful to consider popularity/abundance rates when updating databases, generating new datasets, and carrying out dereplication procedures.

## Figures and Tables

**Table 1:** The number of chemical compounds (millions) in the two databases.

Data Source	ChemSpider [14]	PubChem [15]
Database site	88 (4 August 2020)	103.3 (4 August 2020)
Our estimate	78 ± 2 (25 July 2020)	102.4 ± 0.4 (11 June 2020)

**Table 2:** Statistical errors in estimating one or another proportion of compounds.

Proportion, %	Error, Absolute %	
	ChemSpider	PubChem
<1	<<1	<0.1
1–10	1–2	0.1–0.3
>10–20	2	0.3–0.4
>20–80	2–3	0.4
>80	1–2	0.1–0.3

**Table 3:** Limits of subset ranges of rare and popular compounds.

**Indicator**	**Value Range for the Specified Probability**						**Maximum Value**	
	**Rare Compounds**			**Popular Compounds**			**The Sample**	**The Database**
	0.90	**0.95**	0.99	0.10	**0.05**	0.01		
**СhemSpider**								
Sources	0–3	**0–5**	0–8	≥3	**≥5**	≥9	21	222 ^1^
Vendors	0–3	**0–5**	0–7	≥3	**≥5**	≥7	20	38 ^1^
**PubChem**								
Counts	0–2	**0–2**	0–3	≥2	**≥2**	≥4	15	18
Vendors	0–1	**0–3**	0–5	≥1	**≥3**	≥5	75	98 ^1^
Patents	0–1	**0–3**	0–9	≥2	**≥3**	≥9	23,036	630,628 ^1^
Sources	1–2	**1–3**	1–4	≥2	**≥3**	≥4	20	53 ^1^

^1^ Estimates based on the compounds with the largest number of data sources in ChemSpider at the end of 2020.

## Data Availability

The data presented in this study are available on request from the corresponding author.

## Supplementary Materials

The following are available online, Table S1: ID Numbers, Table S2: The Fifty Most Popular Compounds.
