# Supplementary material for: Statistics of the Popularity of Chemical Compounds in Relation to the Non-Target Analysis
Source: Molecules. 2021 Apr 20;26(8):2394. doi: 10.3390/molecules26082394 (PMC8074313; doi:10.3390/molecules26082394)
Supplement: Supplementary file 1 [file molecules-26-02394-s001.zip › Table 2S. The Fifty Most Popular Compounds.doc.pdf]

Table 2S. The Fifty Most Popular Compounds*

Rating	Name	Structure	Formula	Number of data sources	Class of compounds
1	Niacin	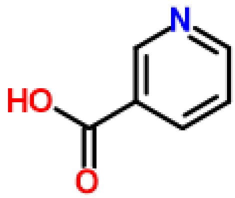	$C_6H_5NO_2$	224	Antilipemic drug, vitamin
2	Fluorouracil	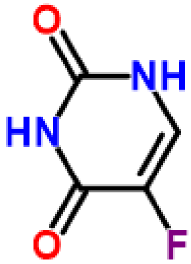	$C_4H_3FN_2O_2$	221	Antineoplastic agent
3	Glycine	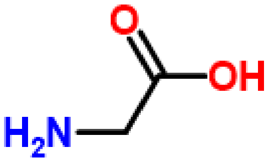	$C_2H_5NO_2$	212	Natural amino acid, antipruritic drug
4	Adenine	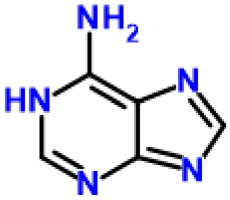	$C_5H_5N_5$	212	Natural nucleobase, drug
5	Indole-3-acetic acid	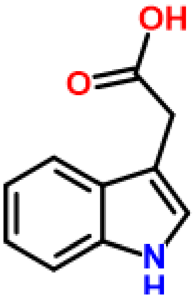	$C_{10}H_9NO_2$	211	Plant growth regulator

Table 2S. The Fifty Most Popular Compounds (continued)

Rating	Name	Structure	Formula	Number of data sources	Class of compounds
6	Paracetamol	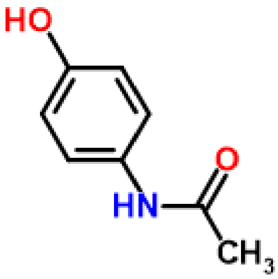	$C_8H_9NO_2$	210	Analgesic, anti-inflammatory drug, antipyretic
7	Allopurinol	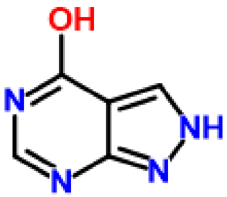	$C_5H_4N_4O$	207	Medicine used to treat gout and kidney stone
8	Trans-resveratrol	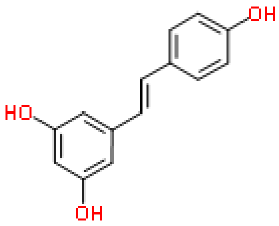	$C_{14}H_{12}O_3$	207	Antidote, antineoplastic agent, antioxidant
9	γ-Aminobutyric acid	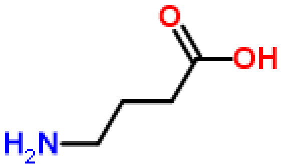	$C_4H_9NO_2$	206	Antihypertensive agent
10	4-Aminobenzoic acid	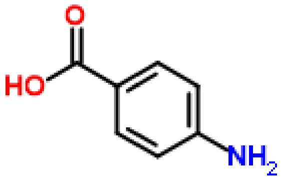	$C_7H_7NO_2$	205	Ultraviolet filter

Table 2S. The Fifty Most Popular Compounds (continued)

Rating	Name	Structure	Formula	Number of data sources	Class of compounds
11	Cytosine	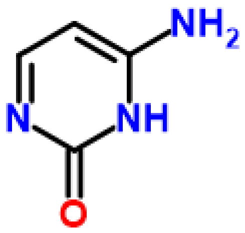	$C_4H_5N_3O$	204	Natural nucleobase
12	Uracil	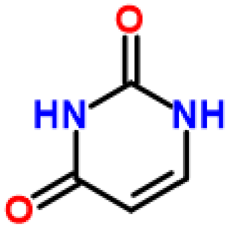	$C_4H_4N_2O_2$	203	Natural nucleobase, antineoplastic agent.
13	4-Aminopyridine	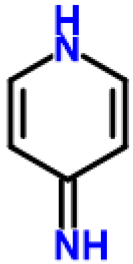	$C_5H_6N_2$	203	Analgesic
14	1,7-Diazaindene	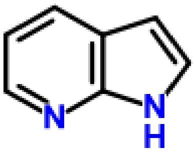	$C_7H_6N_2$	202	Calcitonin gene-related peptide receptor antagonists used in the treatment
15	Benzoic acid	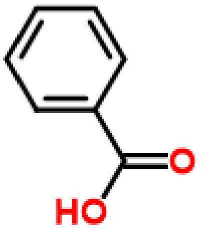	$C_7H_6O_2$	202	Antifungal agent, widespread chemical

Table 2S. The Fifty Most Popular Compounds (continued)

Rat-ing	Name	Structure	Formula	Number of data sources	Class of com-pounds
16	Quinolinic acid	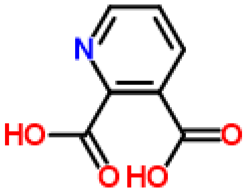	$C_7H_5NO_4$	201	Involved in many disorders and living pro-cesses
17	Ibuprofen	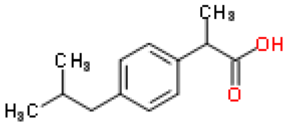	$C_{13}H_{18}O_2$	201	Anti-inflamma-tory drug
18	(E)-Ferulic acid	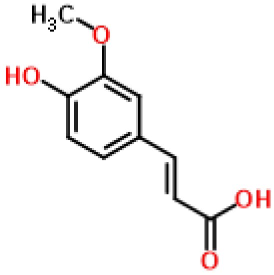	$C_{10}H_{10}O_4$	201	Antihyperten-sive agent
19	Salicylic acid	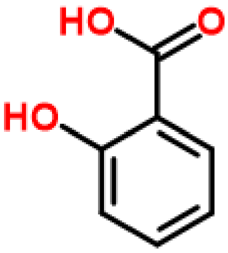	$C_7H_6O_3$	200	Widespread medical use
20	4-Hydroxyben-zal-dehyde	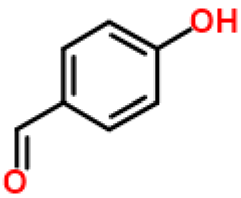	$C_7H_6O_2$	200	Organic reagent

Table 2S. The Fifty Most Popular Compounds (continued)

Rating	Name	Structure	Formula	Number of data sources	Class of compounds
21	Protocatechuic aldehyde	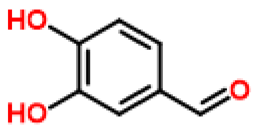	$C_7H_6O_3$	200	Precursor in the biosynthesis, pharmacological agent
22	Fluorouracil	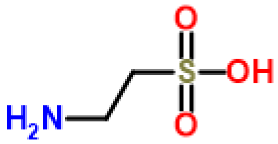	$C_2H_7NO_3S$	198	Antineoplastic agent
23	Tryptamine	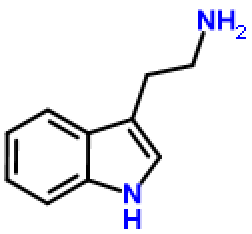	$C_{10}H_{12}N_2$	197	Significant biomedical role, organic reagent
24	8-Hydroxyquinoline	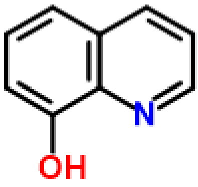	C_9H_7NO	197	Antiseptic drug, disinfectant
25	Quercetin	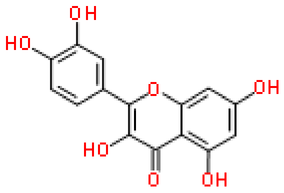	$C_{15}H_{10}O_7$	197	Flavonoid found in many foods and herbs

Table 2S. The Fifty Most Popular Compounds (continued)

Rating	Name	Structure	Formula	Number of data sources	Class of compounds
26	Aciclovir	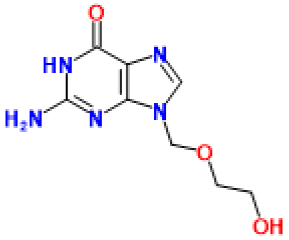	$C_8H_{11}N_5O_3$	196	Antiviral agent
27	4-Hydroxybenzoic acid	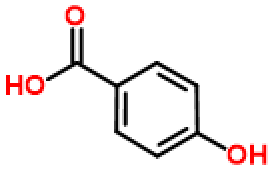	$C_7H_6O_3$	196	Intermediate in synthesis and biosynthesis
28	Melatonin	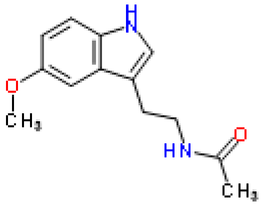	$C_{13}H_{16}N_2O_2$	195	Antioxidant
29	Cinnamic acid	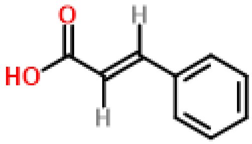	$C_9H_8O_2$	195	Reagent in the synthesis of flavors, dyes, and pharmaceuticals
30	Aminocaproic acid	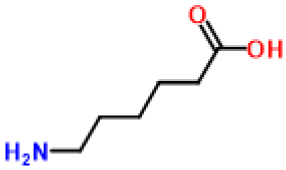	$C_6H_{13}NO_2$	194	Hematological agent

Table 2S. The Fifty Most Popular Compounds (continued)

Rating	Name	Structure	Formula	Number of data sources	Class of compounds
31	Dipicolinic acid	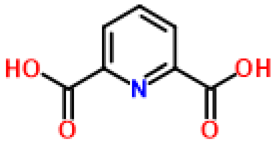	$C_7H_5NO_4$	194	Found in bacterial spores
32	Picolinic acid	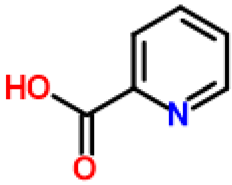	$C_6H_5NO_2$	194	Organic reagent
33	Apigenin	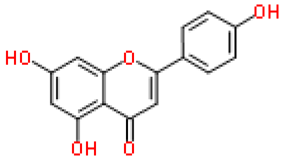	$C_{15}H_{10}O_5$	194	Antineoplastic agent
34	Pyrazinamide	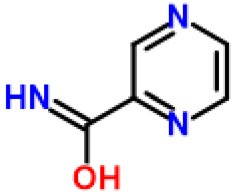	$C_5H_5N_3O$	193	Antitubercular agent
35	Succinic acid	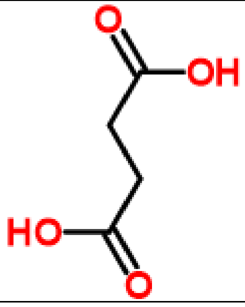	$C_4H_6O_4$	193	Used in food and beverage industry, excipient in pharmaceutical products

Table 2S. The Fifty Most Popular Compounds (continued)

Rating	Name	Structure	Formula	Number of data sources	Class of compounds
36	β-Alanine	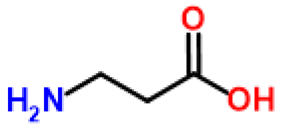	$C_3H_7NO_2$	193	Dietary supplement and other use
37	Gallic acid	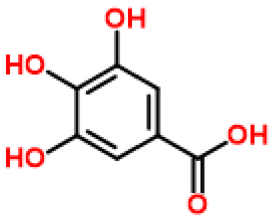	$C_7H_6O_5$	193	Antioxidant; used in tanning, ink dyes, and the manufacture of paper
38	1-Aminocyclopropanecarboxylic acid	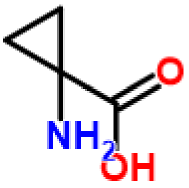	$C_4H_7NO_2$	193	Plant growth regulator
39	Flucytosine	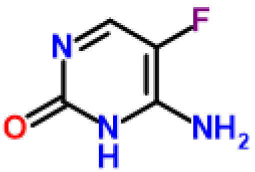	$C_4H_4FN_3O$	193	Antifungal agent
40	Vanillic acid	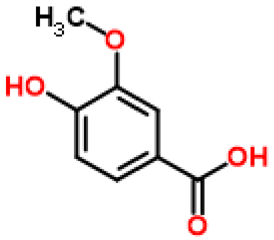	$C_8H_8O_4$	193	Flavoring agent, an intermediate in the production of vanillin

Table 2S. The Fifty Most Popular Compounds (continued)

Rating	Name	Structure	Formula	Number of data sources	Class of compounds
41	Indole-3-butyric acid	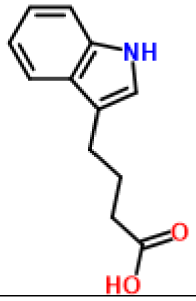	$C_{12}H_{13}NO_2$	192	Plant growth regulator
42	3-Formyl indole	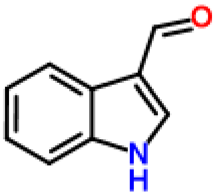	C_9H_7NO	192	Biologically active metabolite
43	Genistein	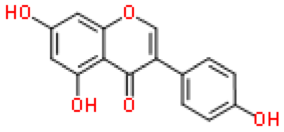	$C_{15}H_{10}O_5$	191	Antineoplastic agent
44	Protocatechuic acid	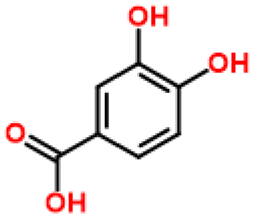	$C_7H_6O_4$	191	Metabolite of antioxidant polyphenols, found in green tea
45	4-Hydroxy-phenyl-acetic acid	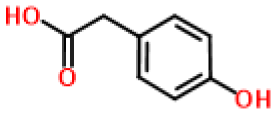	$C_8H_8O_3$	191	Intermediate in organic synthesis

Table 2S. The Fifty Most Popular Compounds (continued)

Rating	Name	Structure	Formula	Number of data sources	Class of compounds
46	Pipecolic acid	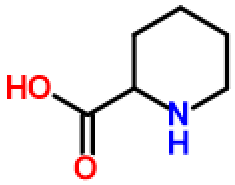	$C_6H_{11}NO_2$	191	Diagnostic marker of epilepsy
47	Mesalazine	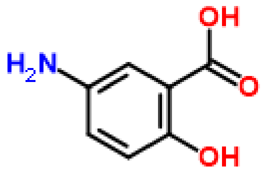	$C_7H_7NO_3$	191	Anti-inflammatory drug
48	Amifampridine	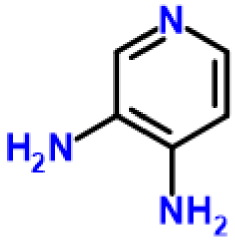	$C_5H_7N_3$	191	Drug in the treatment of rare muscle diseases
49	Thymine	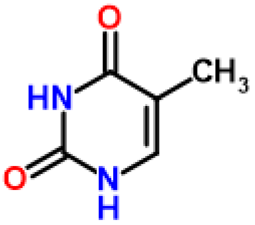	$C_5H_6N_2O_2$	190	Nucleobase
50	5-Bromo-2-pyridinamine	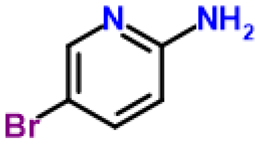	$C_5H_5BrN_2$	190	Organic reagent

* The advanced search of data sources in ChemSpider [14] in April 8, 2021 by selecting all sources. List of 100,1000, or 10000 the most popular compounds are automatically searched. Features of compounds were extracted from this database and the literature cited there.
